# The complete mitochondrial genome of water flea *Ceriodaphnia dubia* (Crustacea: Cladocera) NIES strain

**DOI:** 10.1080/23802359.2023.2241663

**Published:** 2023-08-07

**Authors:** Kyoshiro Hiki, Kenta Oka, Nobuyoshi Nakajima, Haruna Watanabe, Hiroshi Yamamoto, Takahiro Yamagishi

**Affiliations:** a Health and Environmental Risk Division, National Institute for Environmental Studies, Tsukuba, Japan; b Biodiversity Division, National Institute for Environmental Studies, Tsukuba, Japan

**Keywords:** Daphnids, cladocerans, *Ceriodaphnia*, mitogenome

## Abstract

Water flea *Ceriodaphnia dubia* has been widely used for risk assessments of chemicals and environmental contamination. In this study, the complete mitochondrial genome (mitogenome) of this species NIES strain was determined using short-read high throughput and long-read sequencing technologies. The mitogenome of *C. dubia* was 15,170 bp in length and consisted of 13 protein-coding genes (PCGs), 2 ribosomal RNAs (rRNAs), and 22 transfer RNAs (tRNAs). The gene order was identical to the pattern conserved across crustaceans. The complete mitogenome of the NIES strain will serve as genetical reference in ecological risk assessments in Japan, as well as resources for future phylogenetical studies using cladocerans.

## Introduction

**Figure 1.**
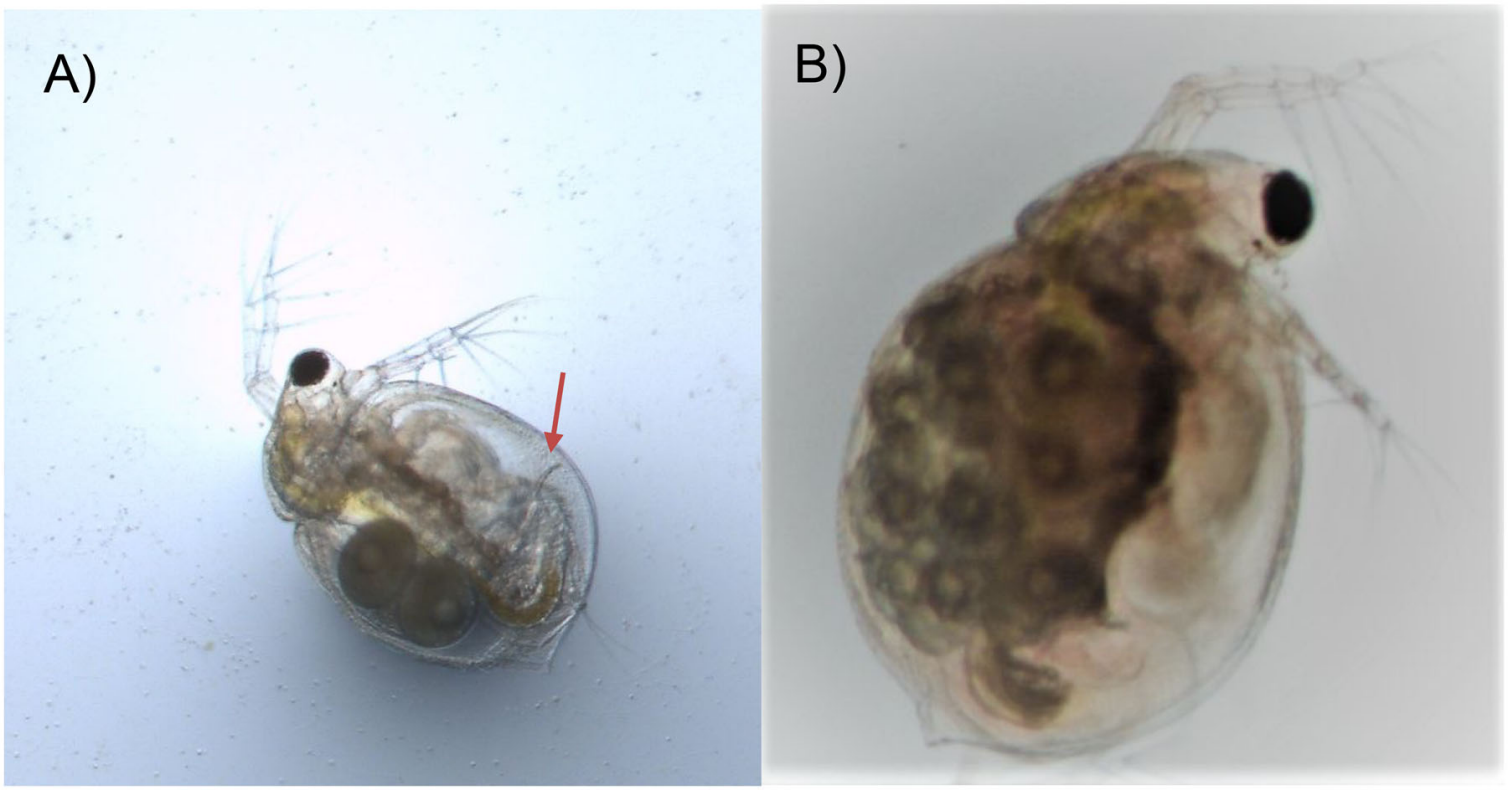
Photos of *Ceriodaphnia dubia.* This species can be distinguished from other related species based on dorsolateral depression between head and rest of body, (A) postadominal claw (arrow), and (B) lack of rostrum and tail spine. The photos were taken by the authors.

The cladocerans are pelagic water fleas and have been widely used in ecotoxicological studies and chemical risk assessments. In particular, *Daphnia magna* Straus 1820, *D. pulex* Leydig 1860, *Ceriodaphnia dubia* Richard 1894, and *Moina macrocopa* Straus 1820 are representative and have been cultured in many laboratories worldwide. National Institute for Environmental Studies (NIES), Japan has established the culture of these cladoceran species (i.e. NIES strains) and provided the NIES strains to laboratories throughout Japan (https://www.nies.go.jp/kenkyu/yusyo/suisei/index.html) for risk assessments of chemicals and environmental contamination (Mano et al. 2010; Hayasaka et al. 2012; Watanabe et al. 2016). Despite its ecotoxicological importance, *C. dubia* (Figure 1) does not have a published complete mitochondrial genome for any strains. In this study, we report the complete mitogenome sequences of *C. dubia*, NIES strain. The complete mitogenome of the NIES strain is beneficial as a genetic resource for future ecotoxicological research as well as to investigate intrageneric phylogenetic relationships.

## Materials

The specimens of *C. dubia* were obtained from laboratory culture that was maintained at the NIES for more than 30 years. The laboratory culture originated from 15 individuals that were transported from the United States Environmental Protection Agency in 1994. Notably, since their initial transportation in 1994, there has been no further introduction from external sources, indicating the uniformity of this lineage.

## Methods

DNA was extracted from whole body using DNeasy Blood & Tissue Kit (Qiagen, Hilden, Germany) and sequenced on an Illumina MiSeq with a paired-end library. The extracted DNA was deposited in the Ecotoxicity Research Section at the NIES, Japan (NIES-202202-CLA1; Kyoshiro Hiki, hiki.kyoshiro@nies.go.jp). The sequence data were processed and assembled using CLC Genomics Workbench version 22.0 following the method described in our previous study (Hiki et al. 2021). Low coverage region between 12S rRNA and *trn*Q was amplified using primers (Forward: 5′-GCTGGCACGATTTTGGTCAAT-3′, Reverse: 5′-GGCATGAGCCCACTAGCTTA-3′) and additionally sequenced using Oxford Nanopore Technologies MinION with SQK-RAD004 kit. We filtered out reads with low-quality scores by NanoFilt version 2.8.0 (De Coster et al. 2018), with parameters of–headcrop 10 and–quality 10. To fill the gap in the draft assembly, we mapped the Nanopore long-reads to the draft assembly and used Pilon version 1.24 (Walker et al. 2014) to integrate these reads with the Illumina short-reads. The accuracy of the assembly was confirmed by visualizing the read coverage depth with integrative genomics viewer (IGV) (Thorvaldsdóttir et al. 2013). The mitogenome was annotated by MITOS 2 webserver (Bernt et al. 2013) and by manual comparison with orthologous genes of other daphnid species. The circular mitogenome was visualized using Proksee (Grant et al. 2023).

Phylogenetic analysis was performed based on 13 protein-coding gene (PCG) sequences in the mitogenome and those of other selected cladocerans within the infraorder Anomopoda. Each amino acid sequence was aligned separately using MAFFT version 7.490 with the L-INS-i option (Katoh and Standley 2013) and then the alignments were filtered using trimAl version 1.4.1 (Capella-Gutiérrez et al. 2009) with the heuristic method. Maximum likelihood-based phylogenetic trees were inferred using IQ-TREE version 2.1.2 (Nguyen et al. 2015) with the ‘–p’ option to allow partition-specific evolution rates and visualized by the ggtree R package version 3.4.4 (Yu et al. 2017). The best-fit model for each PCG was determined by ModelFinder (Kalyaanamoorthy et al. 2017) implemented in IQ-TREE, based on Akaike information criteria.

## Results

**Figure 2.**
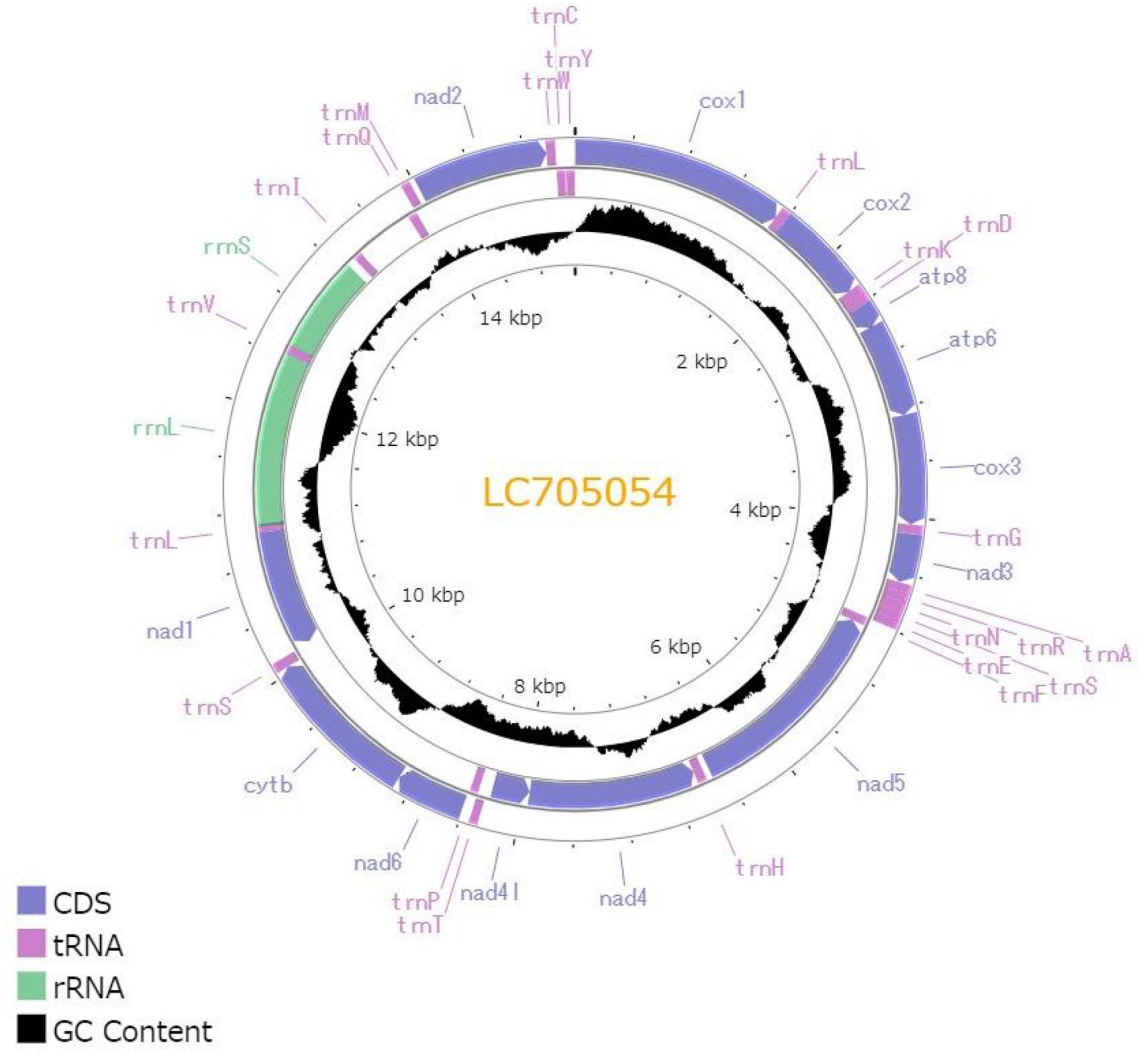
Circular map of the mitochondrial genome of *C. dubia*. Genes outside the circle are encoded on the heavy strand and genes inside the circle are encoded on the light stand. The inner black bars indicate the GC content, and the Middle line represents 50%. Visualization was performed using Proksee (Grant et al. 2023).

**Figure 3.**
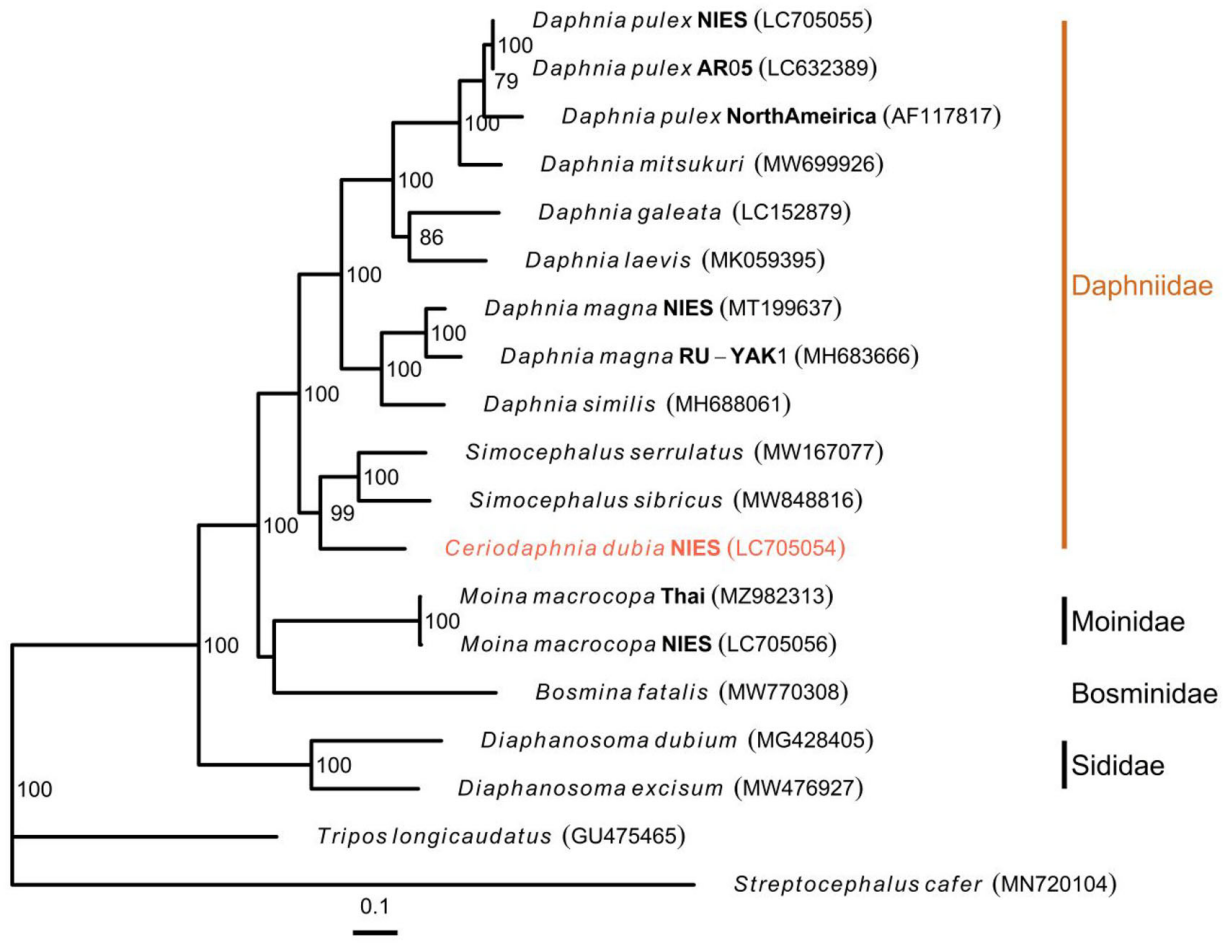
Maximum likelihood Tree based on 13 PCGs in mitochondrial genomes of cladocerans. Red species represent the mitogenomes obtained in this study. *S. cafer* and *T. longicaudatus* were used as the outgroup. Non-parametric bootstrap values (based on 3000 times resampling) are shown at nodes. The scale bar indicates the number of amino acid substitutions per site. The following sequences were used to infer the tree: *Daphnia pulex* LC705055.1, *D. pulex* LC632389.1 (Ohtsuki et al. 2022), *D. pulex* AF117817.1 (Crease 1999), *D. mitsukuri* MW699926.1, *D. galeata* LC152879.1 (Tokishita et al. 2017), *D. laevis* MK059395.1 (Martins Ribeiro et al. 2019), *D. magna* MT199637.1 (Lee et al. 2020), *D. magna* MH683666.1 (Fields et al. 2018), *D. similus* MH688061.1 (Fields et al. 2018), *Simocephalus serrulatus* MW167077.1, *S. sibricus* MW848816.1 (Gu et al. 2021), *C. dubia* LC705054.2 (this study), *Moina macrocopa* MZ982313.1 (Nam et al. 2022), *M. macrocopa* LC705056.1, *bosmina fatalis* MW770308.1 (Wei et al. 2021), *Diaphanosoma dubium* MG428405.1 (Liu et al. 2017), *Diaphanosoma excisum* MW476927.1 (Pan et al. 2021), *tripos longicaudatus* GU475465.1 (Ryu and Hwang 2010), and *streptocephalus cafer* MN720104.1 (Tladi et al. 2020).

The complete circular mitogenome of *C. dubia* NIES strain (INSDC accession numbers: LC705054.2) was 15,170 bp in length (Figure 2). The mitogenome contained typical gene components, including 13 PCGs, two ribosomal RNAs (rRNAs), and 22 transfer RNAs (tRNAs). The order of the genes was completely identical to that reported for the mitogenome of *D. pulex* (Crease 1999). The ratio of A + T nucleotides was 69.5%. All 13 PCGs initiated with the standard start codons (i.e. ATN and TTG). Whereas most of all PCGs terminated with complete stop codons (i.e. TAA and TAG), several PCGs had termination codons consisting of only T: *cox1*, *cox2*, and *nad5*. Putative control region located between 12S rRNA and *trn*Q, and contained two different inverted repeats (47 and 41 bp with GC content of 23.4 and 26.8%, respectively). Although the Illumina reads had low depth of coverage in the putative control region (>20×), likely due to its low GC content, the Nanopore reads provided sufficient depth (>21,000×) (Figure S1), indicating a highly confident assembly. The maximum likelihood-based phylogenetic analysis showed that *C. dubia* was grouped with *Simocephalus* genus (Gu et al. 2021) with a high bootstrap value and that the Daphniidae family formed the monophyletic group within the infraorder Anomopoda (Figure 3).

## Discussion and conclusion

This study presents the first report of the complete mitogenome of *C. dubia* NIES strain. The obtained mitogenome exhibited typical gene components and gene order. Phylogenetic analysis confirmed that *C. dubia* belonged to Daphniidae family. These results, along with the sequence data, establish a valuable genetic resource for the NIES strain of this species, which will contribute to ecotoxicological research in Japan.

## Supplementary Material

Supplemental Material

## Data Availability

Data that support the findings of this study are openly available at INSDC with the accession number LC705054.2, and at Sequence Read Archives (SRA) with the accession number PRJDB13090 (BioProject), SAMD00444315 (*C. dubia*) (BioSample), DRX338201 and DRX338203 (Experiment), and DRR352290 and DRR352292 (Run). The sequences at two different SRA accession numbers were derived from the same DNA sample but from different sequencing runs.
