# Supplementary material for: The complete mitochondrial genome of water flea Ceriodaphnia dubia (Crustacea: Cladocera) NIES strain
Source: Mitochondrial DNA B Resour. 2023 Aug 7;8(8):831–5. doi: 10.1080/23802359.2023.2241663 (PMC10408566; doi:10.1080/23802359.2023.2241663)
Supplement: Supplemental Material [file TMDN_A_2241663_SM5595.docx]

Supporting Information for “The complete mitochondrial genome of water flea *Ceriodaphnia dubia* (Crustacea: Cladocera) NIES strain”

**Author**: Kyoshiro Hiki^a^, Kenta Oka^a^, Nobuyoshi Nakajima^b^, Haruna Watanabe^a^, Hiroshi Yamamoto^a^, Takahiro Yamagishi^a^

^a^ Health and Environmental Risk Division, National Institute for Environmental Studies, 16-2 Onogawa, Tsukuba, Ibaraki 305-8506, Japan

^b^ Biodiversity Division, National Institute for Environmental Studies, 16-2 Onogawa, Tsukuba, Ibaraki 305-8506, Japan

**Corresponding Author**: Kyoshiro Hiki

E-mail: [hiki.kyoshiro@nies.go.jp](mailto:hiki.kyoshiro@nies.go.jp), [hiki.1225@gmail.com](mailto:hiki.1225@gmail.com)

ORCID ID: 0000-0001-7898-9486


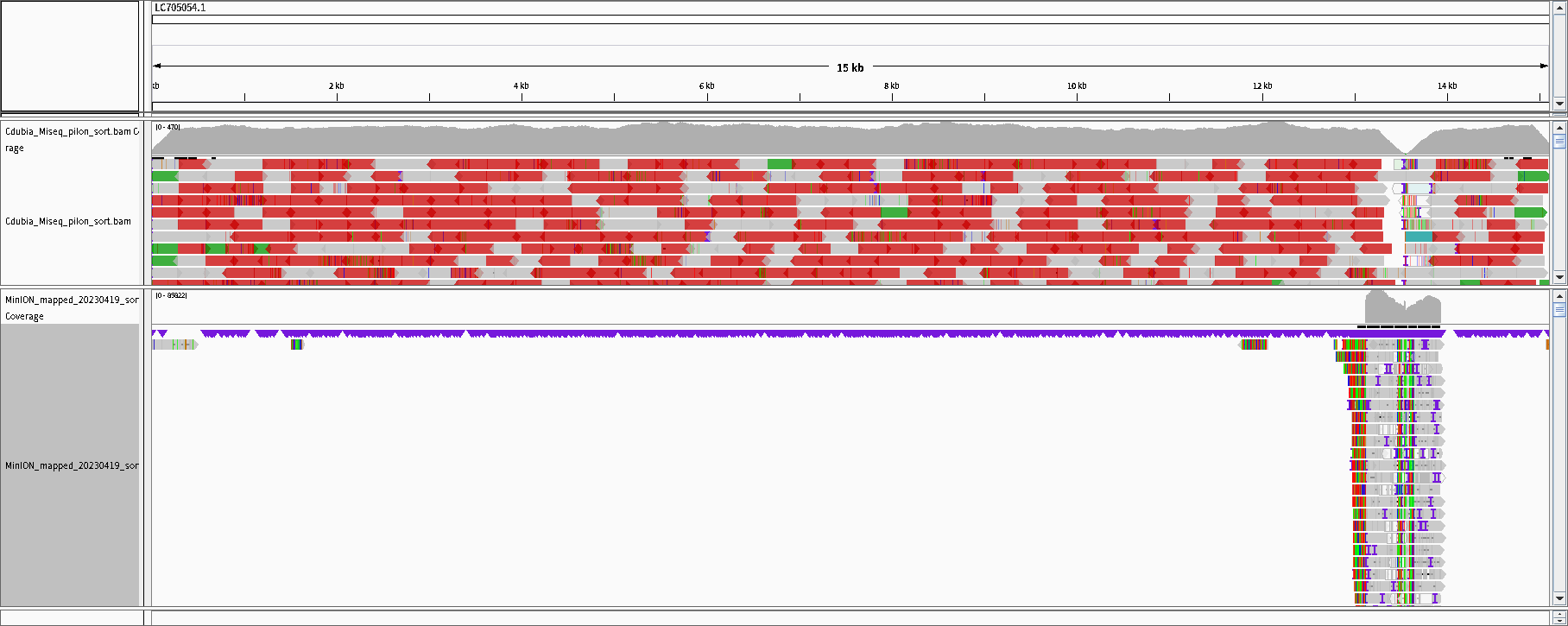


Figure S1. Mapping of Illumina short-reads (upper panel) and Nanopore long-reads (lower panel) to the *Ceriodaphnia dubia* mitogenome. The Illumina short-reads showed extremely low depth of coverage (20×) in the putative control region, while the Nanopore long-reads from the PCR amplicon provided sufficient depth (over 21,000×). Mapping was visualized using integrative genomics viewer (IGV) (Thorvaldsdóttir, Robinson, and Mesirov 2013).

**References**

Thorvaldsdóttir, Helga, James T. Robinson, and Jill P. Mesirov. 2013. “Integrative Genomics Viewer (IGV): High-Performance Genomics Data Visualization and Exploration.” *Briefings in Bioinformatics* 14 (2): 178–92. https://doi.org/10.1093/bib/bbs017.
